# Uncertainty of Standardized Track Insulation Measurement Methods for Stray Current Assessment

**DOI:** 10.3390/s23135900

**Published:** 2023-06-25

**Authors:** Sahil Bhagat, Jacopo Bongiorno, Andrea Mariscotti

**Affiliations:** 1 DITEN, University of Genova, 16145 Genova, Italy; 5025527@studenti.unige.it; 2 RINA Services S.p.A., 16138 Genoa, Italy; jacopo.bongiorno@rina.org

**Keywords:** DC power systems, guideway electric transportation systems, stray current, test methods, uncertainty

## Abstract

Stray current is a relevant phenomenon in particular for DC electrified transportation systems, affecting track and infrastructure within the right of way and other structures and installations nearby. It worsens with time and the level of protection depends on timely maintenance, as well as correct design choices. The assessment of track insulation is the starting point for both stray current monitoring systems and at commissioning or upon major changes. Standardized methods (ref. EN 50122-2 or IEC 62128-2) have been almost unchanged in the last 20 years but suffer from accuracy issues and variability due to parameters and conditions not under the operator’s control. The uncertainty of test methods is increasingly important now that contractual specifications require a high level of insulation for new systems. A critical discussion and analysis of the sources of variability and practical constraints is proposed, followed by an evaluation of uncertainty, with the objective not only to assess the accuracy of the provided results, but also to foster research on innovative, more flexible and accurate methods.

## 1. Introduction

All electrified transportation systems (ETSs) of the guideway type are affected by return current leakage from the guiding track into the soil, coupling to structures nearby. Examples of victims are sleepers and the track bed [1,2,3], platform screen doors and other metallic parts at platforms [4], viaducts, bridges and building foundations [5,6], pipelines and reservoirs [7,8,9], etc., as well as corrosion of power earthing systems and saturation of transformers [10,11,12].

In general, the effect of current flow through the interface of a metal with an electrolyte solution (such as the soil itself, or cement for concrete structures) causes corrosion, affecting the solidity and durability of said structures. The first victims are the sleepers and rail fasteners [13,14,15]: in the case of a local insulation loss, they may become a hotspot, with a significant increase in current density, although the overall track leakage may be acceptable over a longer length.

For this reason, preferred verification methods should be able to operate on a local basis, that is, for short enough track sections, providing a valuable indication for rapid visual inspection and repair. However, they should also be easily applicable to longer stretches, favoring rapid diagnosis of a long line, at least as a periodic preliminary check.

Track current insulation phenomena, apart from the spatial dimension, develop through time, with testing, monitoring and evolution of insulation degradation having different time scales:

- from seconds to minutes, if applying test signals in off-service conditions: time intervals of seconds are necessary for the polarization of electrolytes in the test circuit to take place, after which, test quantities can be measured with care to reject external noise with sufficiently long observation times;
- from hours to weeks, if using track electric quantities during train service:
- from days to years, considering the normal evolution of track insulation degradation, with aging of insulating materials, pollution of surfaces, stagnation of water, etc.

The relevance of stray current assessment, in general, is proven by the consequences of corrosion: weakening of structures within the right of way and nearby, impairment of track stability, spillage and breakage of pipes and reservoirs, more onerous repairs and corrective maintenance rather than normal preventive operation.

A significant modeling and simulation effort has developed through the years in order to understand the coupling mechanisms and to globally address the problem with suitable design choices and provisions [16,17,18,19,20]. Prevention and compensation of stray current is, in fact, taking place by means of various systems: optimization of traditional track and transit systems [21,22], passive stray current collection systems [23], traditional track voltage limitation [4,24], active track potential control and redistribution of traction current [25,26].

Stray current monitoring systems are gaining popularity [27] as they provide feedback on the health status of an important asset, although the interpretation of collected data and identification of necessary actions with the right timing are still complex problems that are unsolved [28].

The measurement of voltage and current quantities at track, substation negative and stray current collection is as accurate as the combination of probes used and the sampling channels. However, stray current evaluation using such approaches necessitates initial tuning (and, possibly, periodic verification) that depends on the assessment of current track insulation: sources of variability beyond instrumental uncertainty, including site conditions, are, thus, considered in this work, focusing on standardized methods.

In particular, the humidity conditions and the wet status of the track are not explicitly indicated nor discussed in the standards. A large deal of track insulation variation can be ascribed to the water percentage: a water film on the surface of the track and fastener insulating elements can compromise the otherwise good insulation level provided by the volumic insulation resistance of polymeric materials. For an increased amount of water, to the extent of completely wet fasteners and rail base, any insulating provision is compromised and the measured performance is that of electric conduction through water. For example, a 6.6 mS/km track wetted by water jet increased its conductance 15 times, returning to an intermediate value five times larger than the dry reference value after 30 min on a dry day at 28 °C.

Track insulation measurement is guided by the standard EN 50122-2 [29] (equivalent to the IEC 62128-2 that is still in its 2013 version [30]) that distinguishes three methods:

- method A.2, track insulation with civil structure: the important point that makes this less invasive compared to method A.3 is that the running rails are continuous and do not need to be sectioned; however, the test setup is more complex, with more measured quantities involved and an overall worse accuracy. The evaluation of uncertainty and optimal test conditions is discussed in Section 2;
- method A.3, track insulation without civil structure: the presence or absence of the civil structure is not the relevant point here, as the running rails must be sectioned to the desired length, either by cutting them or exploiting the presence of insulating rail joints; this is a more accurate method and should be preferred whenever possible, especially for high track insulation values;
- method A.4, lateral voltage gradient method in open area sites: this method measures the voltage gradient in the soil caused by running trains, with the field laterally extending from the tracks at two points at different distances; it is suitable for large open areas, but necessitates access to soil as homogeneous as possible and may, thus, be disturbed by buried structures and installations, such as in an urban context.

With contractual specifications requiring track insulation levels of the order of 100 Ωkm or better, the uncertainty of measurement methods is negatively affected at such low track leakage current levels, as we will see in the following, with some methods performing better than others.

Apart from the metrological aspects, such as variability and uncertainty, the three methods have different impact and cost in terms of organization and preparation, including the necessary operations to set up the test and to bring back the system in the original conditions. These, in fact, are the real cost-driving factors and, in general, methods that can be overlapped to the track without affecting its structural integrity should be preferred (in our case, methods A.2 and A.4). In addition, the overall time duration to fit within the engineering hours and the compatibility with commercial traffic for systems already in operation are two other relevant aspects: method A.2, of course, cannot be used in the current implementation in the presence of trains, and method A.3 is absolutely not compatible with train runs, whereas method A.4 exploits the normal line traffic as the test signal (and provides track voltage information as well).

The purpose of this work is to analyze and discuss three methods for track insulation assessment that are reported in the Annex A (informative) of the EN 50122-2 standard [29,31]. Their description has been unchanged for more than 20 years and the test experience of several years, including discussions during test campaigns, has uncovered various points that are vague, insufficiently detailed, and, in some cases, have had a significant impact on the quality of the results and their uncertainty.

Section 2, Section 3 and Section 4 consider the three methods separately, discussing the setup and equations, providing practical considerations and identifying relevant factors, and then proceeding to the estimation of variability and uncertainty. Each section provides numerical examples and analyzes experimental data from past test campaigns. The outcomes are then summarized and discussed by comparing the three methods in the conclusions in Section 5.

## 2. Method A.2: Continuous Track, Line Closed to Traffic

### 2.1. Method Description and Setup

**Figure 1:**
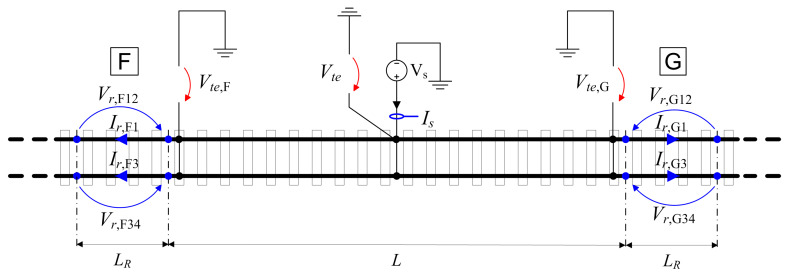
Sketch of the method A.2 setup for a track insulation measurement: the current measuring circuits in blue, the voltage measuring circuits in red.

The method basically achieves the estimation of the leaking current in the track section under measurement (from now on, simply “track section”) by subtracting the rail current flowing outside the track section (indicated by $I_{r,F1}$, $I_{r,F3}$ for the leftmost position and $I_{r,G1}$, $I_{r,G3}$ for the rightmost position) from the applied source current $I_{s}$. The setup, annotated with the relevant electrical quantities, is shown in Figure 1.

The $I_{s}$ intensity depends on the used voltage level $V_{s}$ and the overall resistance-to-earth of the entire system, that is, of the tracks that can be considered electrically continuous from the point of injection of the test current. It is evident, then, that the $I_{s}$ current does not change with the location and injection point. In addition, a very well-insulated system will sink a low test current anyway. The use of higher test voltages of course increases the test current intensity, but this is usually not performed only for this purpose, but rather to provide a test level that is much larger than the internal voltage barriers due to oxidized surfaces and electrolytes, ruling them out as a major source of error. To this end, polarity reversal may be used, canceling to some extent such offset voltages. A 50 V test is a common choice, as it does not pose serious problems of electrical safety.

The current flowing along the rails in the measured track section causes a voltage drop (indicated as $V_{r,F12}$, $V_{r,F34}$,$V_{r,G12}$, $V_{r,G34}$ in Figure 1) that, for moderate current intensity, is negligible, but in a general perspective should be included.

The rail current leaving the track section is measured either by direct measurement or by means of the voltage drop on the rails themselves (as suggested by EN 50122-2). The former approach is hindered by the lack of such large openable DC current sensors (Rogowski coils work in AC). The latter approach is commonly used and relies on the measurement of a small voltage drop of the order of some mV. The voltage drops $V_{r,F12}$, $V_{r,F34}$, $V_{r,G12}$, $V_{r,G34}$ are given by the local rail resistance $R_{r,F1}$, $R_{r,F3}$, $R_{r,G1}$, $R_{r,G3}$ multiplied by the flowing current, so that the latter can be estimated by measuring or assuming the rail resistance values.

(1) $$I_{r,F1}=\frac{V_{r,F12}}{R_{r,F1}}\;\;I_{r,F3}=\frac{V_{r,F34}}{R_{r,F3}}\;\;I_{r,G1}=\frac{V_{r,G12}}{R_{r,G1}}\;\;I_{r,G3}=\frac{V_{r,G34}}{R_{r,G3}}$$

The precaution advised is not to include welded points or fish plates (bypassing an insulating rail joint, IRJ) in the rail segment that is used to measure each respective voltage drop. As indicated by EN 50122-2, such welding points could add up to 5% of the longitudinal rail resistance, impacting on accuracy. In addition, the rail length $L_{R}$, across which the voltage drop is measured, is prescribed to be 10 m [29]. When the intensity of the test current $I_{s}$ is not large (for example, due to an overall well-insulated system) and the voltage drop signal intensity is not sufficient, a doubled length brought to 20 m is a sensible compromise to increase it. In general, the rail resistance is taken from datasheets or measured at the location where tests are carried out, and the resulting per-unit rail resistance value $R_{r}$ is unique for all four voltage drop measuring points.

(2) $$R_{r,F1}=R_{r}L_{R}\;\;R_{r,F3}=R_{r}L_{R}\;\;R_{r,G1}=R_{r}L_{R}\;\;R_{r,G3}=R_{r}L_{R}$$

For DC and very low frequency, the relevant rail resistance is the DC value, that can be assumed, in general, to be between about 33mΩ/km to 40 mΩ/km, as documented in [32]. The most commonly used rails for, e.g., metro applications, are of the UIC 54 and UIC 60 types, with different hardness levels, providing resistance of the order of 36 mΩ/km and 33 mΩ/km, respectively. It is apparent that, with a rail current flowing outside the test section of, e.g., 10 A, the expected voltage drop is 3.6 mV and 3.3 mV, respectively, over a 10 m voltage drop measuring rail segment.

The final complete formula for determination of the track-to-earth resistance $R_{te}$ is given by

(3) $$R_{te,A2}=\frac{(\delta V_{te}+\delta V_{te,F}+\delta V_{te,G})/3}{I_{s}-\left(I_{r,F1}+I_{r,F3}+I_{r,G1}+I_{r,G3}\right)}$$

where δ voltage quantities are understood as the difference between the on and off condition value, for example, $\delta V_{te,F}=V_{te,F}\left(\mathrm{on}\right)-V_{te,F}\left(\mathrm{off}\right)$. Multiplication by *L* expressed in km gives the per-unit insulation resistance to compare with the limits.

It is observed that EN 50122-2 [29] reports this formula incorrectly, as the shown test setup is for the whole track (so, for estimate of the track-to-earth insulation), but the current quantities are only two, taken for one rail only.

### 2.2. Practical Factors

Voltage drop readings are generally affected by a significant common-mode disturbance (any time the signal is read by a non-insulated voltage probe), as well as the pre-existing flowing current, e.g., at the utility frequency ( 50 Hz) or fluctuating at low frequency. The reason is the unavoidable influence of external sources, in particular, if the system is already energized and operating, so that substations during engineering hours still have the negative connected to the track. The unavoidable difference in the potential of the utility earth at different locations causes a current flow along the tracks.

In addition, the rail current marked in Figure 1 is, ideally, the one flowing along the short rail section of length *d* that provides the indirect voltage drop measurement. In reality, the current entering such a section flows along the rails and leaks transversely at the same time, for which, the larger the *d*, the larger the difference between these two current values entering and exiting the measurement section. The measurement error can be minimized by assigning to each $I_{r,X}$ the average value within the section as the voltage drop measurement implies, and, thus, using an effective length of the section under measurement equal to L+d, rather than *L*.

The use of rail current probes would solve the problem of the small longitudinal voltage drop across the rails and would provide a floating signal that rejects the common-mode rail potential. Possible probes include Hall effect and fluxgate probes that are large enough to embrace the rail section or Rogowski coil, provided that the test is carried out with a low-frequency AC signal, but not a pure DC component.

In addition, if carrying out the test with a DC source, offsets are quite relevant, being of the same order of magnitude as the expected voltage drop (see the previously estimated 3.3 mV to 3.6 mV).

**Figure 2:**
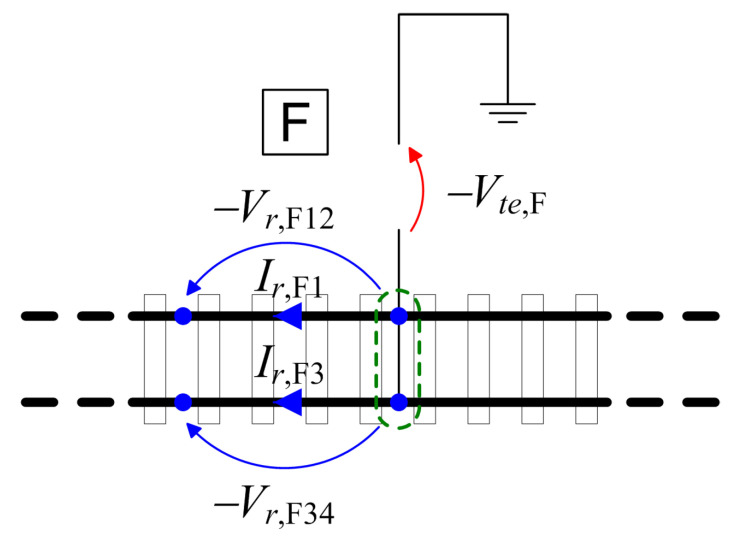
Modification in method A.2 to the rail voltage drop measuring terminals and to the track-to-earth voltage measuring terminal in order to obtain a unique potential reference node (circled in green) and avoid ground loops.

A general optimized arrangement for the classical setup (using voltage probes, as shown in Figure 1) requires a short-circuit connection (cross bond) between rails, creating a reference node at the remote (A or B) location to which both voltage drop readings are referred (shown in Figure 2).

Galvanic isolation and rejection of the common mode can also be achieved using differential voltage probes that, however, introduce a signal reduction (such as a 1:10 factor) and additional noise (being active devices). The use of rail current probes instead would solve the problem *tout court* providing a cleaner floating signal: Hall-effect or fluxgate probes of this size do not exist to the authors’ knowledge, so that a Rogowski coil should be used that requires AC test signals. Some Rogowski models have, in reality, a frequency response extended down to a few Hz and good droop capability, so that an alternating DC voltage could be exploited.

Using instead a real AC signal (such as that provided by an amplifier) makes the method more complex (signal generator and amplifier to add), but provides a clean current measurement. It is only a formal issue, accepting that the track insulation resistance values measured with a pure DC current or a low-frequency AC signal are comparable. To this end, two points need to be checked regarding the equivalent circuit of a running rail.

- The longitudinal impedance of a running rail is comprised of an internal inductance term and an AC resistance term, both accounting for AC effects, such as the skin effect and hysteresis, and related losses [33,34]; values for low current (< 50 A) amount to about 0.8 μH/m and 100 μΩ/m, with the former contributing less than 25 μΩ/m of the inductive reactance at 50 Hz.
- The stray capacitance of a running rail amounts to less than 10 nF/m (obtained by multiplying by 2 the values shown in [35]), providing more than 300 kΩ of capacitive reactance, easily shunted by the transversal track insulation term considered here.

We may conclude that the equivalent circuit of a running rail in AC at low frequency does not differ from that at DC for the purpose of determining the track-to-earth insulation resistance, with the exception that the longitudinal resistance (and impedance) are larger and cannot be used any longer reliably for the determination of the flowing current. The measurement of the flowing rail current could then be achieved by using Rogowski coils that ensure a complete rejection of common-mode signals and a more favorable signal-to-noise ratio. Another method of rail current measurement that allows train circulation, and can be a semi-permanent installation, is by means of close-up sensors, based on the inductive effect [36], with arrays of such elementary sensors for better reconstruction [37], or alternative methods, such as the Hall or magneto-resistive effect [38].

The locations A and B and the central point of injection of the test signal could be separated by a hundred meters, as well as 1 km or so, so that connecting all channels to a single data acquisition system is a problem, not only for the length of the necessary cables, but for the consequential noise pickup of such cables. Separate measuring stations are preferred, necessitating, as a matter of fact, more personnel and instrumentation.

### 2.3. Variability and Uncertainty Analysis

Variability is considered by listing the external factors that have a significant influence on the results and that should be accounted for in the uncertainty analysis. The identification of such factors and their spread is, thus, a necessary initial step for the uncertainty analysis that focuses on (3).

The rail current $I_{r,X}$ (with “X” standing for F1, F3, G1, or G3) is estimated by measuring the rail voltage drop over the rail section of length $L_{R}$ and applying (1). The uncertainty is then

(4) $$u\left\{I_{r,X}\right\}=\sqrt{{\left(u\left\{V_{r,X}\right\}\right)}^{2}+{\left(u\left\{R_{r,X}\right\}\right)}^{2}}$$

where the uncertainty of the voltage reading $u\{V_{r,X}\}$ is derived directly by the employed instrumentation, and that of the rail resistance $u\{R_{r,X}\}$ is related to its variability, and to the availability of measured values for the specific system and track section, or the use of tabular data.

For $u\{V_{r,X}\}$, it is observed that the reading scale is quite low, of the order of a few mV, whereas the track voltage readings are in the more favorable range of tens of V. The measuring multimeter/data logger must be carefully selected as apparently good items of equipment might have quite different performance in the mV range. An overview is provided in Table 1.

From the uncertainty estimates above, it is clear that extreme care must be adopted for the voltage drop measurement and suitable instrumentation must be selected. Medium-performance portable multimeters, such as Fluke 117, are clearly inadequate, and a high-performance multimeter, such as the H29S, barely achieves the minimum necessary performance (about 8% of uncertainty budgeted for the rail current measurements and the remaining 6% for the three track voltage $V_{te,X}$ measurements and the test current $I_{s}$ measurement). Specialized portable data acquisition systems, such as Minilog2, achieve a satisfactory target performance of about 1%, whereas generic ones (e.g., the National Instruments card) achieve the same performance as a high-performance multimeter.

The uncertainty of the rail resistance values is not influenced by the instrumental uncertainty of the rail resistance measurement, but by the variability between rails of the same section and, in general, of different sections (from different production batches or different manufacturers). The problem is discussed in [32], where various examples of experimentally determined values are provided as well.

The expression at the denominator of (3), where the current leaking within the track section is determined by the difference of the injected test current $I_{s}$ and the “escaping” rail currents (flowing outside the section), is inherently exposed to measurement errors, especially for well-insulated tracks. Let us assume that we test with a test voltage $V_{s}=50\;V$, resulting in a test current $I_{s}=10\;A$, a track section of 100 m of a well-insulated system, such as one with 100 Ωkm insulation, resulting in $R_{te}=1\;k\Omega$ for the measured section. The expected leaking current is approximately given by $I_{l}^{*}=V_{s}/R_{te}\approx 50\;\mathrm{mA}$. The four escaping currents ($I_{r,F1}$,$I_{r,F3}$, $I_{r,G1}$, $I_{r,G3}$) must, thus, give about 9.95 A in total, so about 2.5 A each, or, in the extreme case of a measurement taken near the beginning of the line, two rail currents will be at about 5 A and the other two approximately zero. A 1% uncertainty for each of these currents will cause a measurement error $\varepsilon_{Ir,X}\approx 50\;\mathrm{mA}$, that is exactly in the range of the target value of the leaking current. It is easy to see that a 200% measurement error can be reached under the reasonable assumption of random combination of the four error terms compared to $I_{l}^{*}$. The method, thus, provides acceptable accuracy only if:

- a long track section is tested: a 10-times longer track ( 1 km) will provide a 10-times larger $I_{l}^{*}$ and the resulting errors will, this time, be about 20% (large, but acceptable);
- a track with poor insulation is tested: similarly, an insulation level of only 10 Ωkm will provide a similar distribution of the errors, so a 10-times larger $I_{l}^{*}$ will again reach an uncertainty of the order of 20%.

The uncertainty of (3) can be estimated by formally calculating the propagation of uncertainty for each of the relevant quantities. The four rail current terms at the denominator and the three track voltage terms at the numerator have identical effects and may be calculated only once.

The current terms at the denominator form a difference that is handled by using the absolute and not the relative error, so that they are not ready to be expressed in terms of uncertainty. For a difference C=A−B, the following expression holds var[C]=var[A]+var[B]. It is easy to see that *A* indicates $I_{s}$ and *B* indicates $\left(I_{r,F1}+I_{r,F3}+I_{r,G1}+I_{r,G3}\right)$, and their difference may be indicated for simplicity as δI. These expressions, however, can be manipulated based on the assumption that the result is small, as $I_{s}$ and $\left(I_{r,F1}+I_{r,F3}+I_{r,G1}+I_{r,G3}\right)$ are almost equal (quite true for a well-insulated system): the difference is, thus, expressed as a small multiplying coefficient *k* of the half sum, or of either of the two terms with an acceptable degree of approximation.

(5) C=A−B=k(A+B)/2≈kB

where the rightmost equality is justified by the fact that the uncertainty of the measured $\left(I_{r,F1}+I_{r,F3}+I_{r,G1}+I_{r,G3}\right)$ is much larger than that of $I_{s}$ when the used methods are the rail voltage drop and current clamp (or shunt), respectively.

In practice, with a test current $I_{S}=20\;A$ and a test voltage $V_{S}=50\;V$, a track with a good insulation level of 100 Ωkm would have leakage of only $I_{l}=V_{S}/200\;\Omega =50\;\mathrm{mA}$ for a 200 m-long track section. The value of *k* would then be $k=I_{l}/I_{S}=0.25\%$, smaller or comparable to the accuracy of the used instrumentation, as estimated in Table 1. A longer test section, e.g., 1 km, would provide k=1.25%.

The three voltage terms at the numerator follow a similar rule, that is, the summation is managed by using the absolute error, or, in other words, the dispersion or variance, and not its relative (or normalized) version. Assuming that they are measured with the same instrumentation (such as an identical multimeter or different channels of the same data acquisition system), their variances are identical, so that the resulting variance of the average $V_{te,\mathrm{avg}}$ is one third of them: $\mathrm{var}\left[V_{te,\mathrm{avg}}\right]=\mathrm{var}\left[V_{te,X}\right]/3$.

It is possible, thus, to estimate the uncertainty considering (3) as a pure ratio, having introduced the factor *k*:

(6) $$\mathrm{var}\left[R_{te,A2}\right]\approx \sqrt{{\left(\frac{\partial R_{te,A2}}{\partial V_{te,\mathrm{avg}}}\right)}^{2}\mathrm{var}\left[V_{te,\mathrm{avg}}\right]+{\left(\frac{\partial R_{te,A2}}{\partial \delta I}\right)}^{2}\mathrm{var}\left[\delta I\right]}$$

The two terms then correspond to:

(7) $$\frac{\partial R_{te,A2}}{\partial V_{te,\mathrm{avg}}}=\frac{1}{\delta I}=\frac{R_{te,A2}}{V_{te,\mathrm{avg}}}\;\;\frac{\partial R_{te,A2}}{\partial \delta I}=-\frac{V_{te,\mathrm{avg}}}{{\left(\delta I\right)}^{2}}=\frac{R_{te,A2}}{\delta I}$$

The uncertainty of $R_{te,A2}$ is then given by

(8) $$u\left\{R_{te,A2}\right\}\approx \sqrt{{\left(u\{V_{te,\mathrm{avg}}\}\right)}^{2}+{\left(u\{\delta I\}\right)}^{2}}=\sqrt{{\left(u\{V_{te,\mathrm{avg}}\}\right)}^{2}+{\left(k/2\;u\{I_{r,X}\}\right)}^{2}}$$

The reciprocal of the factor *k* is the amplification effect observed above for the resulting uncertainty.

## 3. Method A.3: Sectioned Track, Line Closed to Traffic

### 3.1. Method Description and Setup

This method requires the interruption of the longitudinal electrical conductivity of the rails at the two points that define the measured track section of length $L_{t,A3}$. Then, measuring the electrical insulation between a rail segment and the earth is a straightforward volt-amperometric measurement: a voltage $V_{s}$ is applied between the rail and the earth at one of its ends. The measurement of the flowing current $I_{s}$ must be accompanied by the measurement of the rail-to-earth voltage, not only at some intermediate preferred position $V_{te,A3}\left(P\right)$ (EN 50122-2 indicates a minimum distance of 50 m from the injection point, but not a maximum one), but also at the opposite end, in order to estimate the voltage drop along the rail or track $V_{te,A3}\left(Q\right)$. The difference between the two voltages is required by EN 50122-2 [29] to be less than 10%, but nothing is said on how to remediate in cases where this requirement is not fulfilled. The effect on the resulting track insulation is discussed below in Section 3.2.

**Figure 3:**
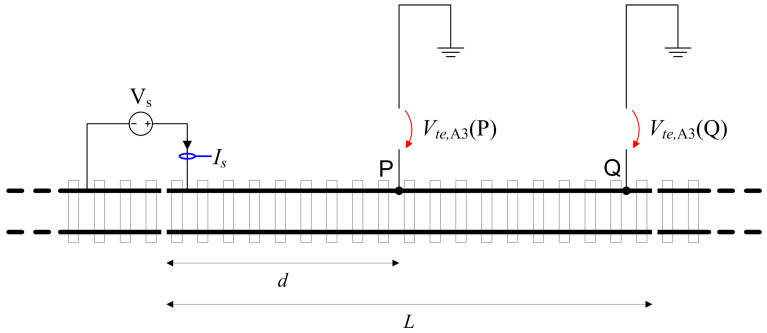
Sketch of the method A.3 setup for a rail insulation measurement: the current measuring circuits in blue, the voltage measuring circuits in red.

The method could be applied to a single rail or a whole track. The latter is a necessity in the presence of frequent rail-to-rail bonds, including coupling bonds for track circuits that at DC are short-circuit connections as well. The setup is shown in Figure 3 for the measurement of the whole track.

EN 50122-2 proposes a simple relationship, such as

(9) $$R_{te,A3}=\frac{V_{te,A3}\left(P\right)}{I_{s}}$$

where *L* is expressed in km. Again, multiplication by *L* expressed in km gives the per-unit insulation resistance to compare with the limits.

This method is more accurate than method A.2, with a minimum number of quantities involved and no need to estimate the current flowing in each rail. Assuming again a test voltage up to 50 V, for rail segments of some hundreds of meters, the flowing test current ranges from some mA up to a fraction of A for the most common track insulation values and can be conveniently measured with an amperometer (multimeter). The method is exposed to some variability as a consequence of variable or not well-specified parameters (earthing resistance of the test supply, distance *d* of the intermediate voltmetric terminal, overall length of the track section *L*) that are reviewed in Section 3.3 based on results in [39,40,41].

### 3.2. Practical Factors

The earthing of the power supply providing the test voltage $V_{s}$ and the earth reference for the voltmetric terminals at P and Q can be implemented in various ways based on practical convenience:

- an earth electrode may be used driven into the soil at a convenient distance from the tested track (EN 50122-2 requires 30 m minimum); the reason for such distance is avoiding distortion of the electric field in the soil; the earthing resistance is quite limited anyway, for which, even in good soil, values lower than about 50 Ω are difficult to achieve, so that this earthing system is suitable for the voltmetric terminals, but not for the test supply;
- using the remaining part of the system before the injection point, earthing the test supply with a resistance $R_{0}$ usually of some Ω; with systems of limited length or still under construction, instead, $R_{0}$ reaches too high values; the influence of this parameter was evaluated in [39] and is considered later in Section 3.3;
- earthed parts, such as cable trays, sharing the earthing resistance of the power distribution system, usually of the order of 1 Ω or less, can be used for both purposes (earthing the power supply and providing a reference for voltmetric measurements);
- the concrete structure supporting the track, if provided with reinforcement, cannot be used, being too close to the track under test.

Since, in many cases, the electrical isolation of the track section is achieved by pre-existing IRJs, choosing a too short section length (with a larger insulation resistance value) exposes the results to the influence of the far-from-ideal isolating performance of the IRJ. For example, a well-insulated system with $R_{te}=100\;\Omega \mathrm{km}$ amounts to 1 kΩ for a section length of 100 m; the measurement could be compromised by an IRJ with insulating resistance of the order of 10 kΩ (still barely acceptable in terms of the standard).

### 3.3. Variability and Uncertainty Analysis

This method, as the most accurate, was assessed for its variability in [39,40,41].

The test should be performed by measuring $V_{te}$ in on and off conditions, so compensating for pre-existing potentials. It was observed in [39] that practical measurements show a significant rapid decay of the potential during depolarization and that a lack of synchronization of a few seconds could cause a significant error. In fact, EN 50122-2 does not clarify the procedure to adopt to take the off-condition reading: for insulating the rail joint efficiency only, it specifies “directly after the switching off”; however, this does not clarify if we are speaking of a fraction of a second or some seconds. The off potential is supposed to be subtracted from the on reading, aiming at compensating extraneous voltages, but the rapid decay (with a steep slope) implies a significant error for timing errors in the first second or so. A better technique is that of polarity reversal that compensates for offset voltages and other bias voltages.

The effect of rail resistance was also considered on the two terminal voltages $V_{te,A3}\left(P\right)$ and $V_{te,A3}\left(Q\right)$, and on the estimated track insulation. Considering the value of $I_{s}$, as determined approximately by $I_{s}=V_{s}/R_{te,A3}$, a worst-case scenario of maximum voltage (50 V) and lowest track insulation (2 Ω/km) would bring this to $I_{s}=25\;A/\mathrm{km}$. Recalling the track resistance of the order of 16.5 mΩ/km to 18 mΩ/km, this causes a maximum longitudinal voltage drop of 0.45 V/km, that is less than 1% of the applied voltage. The requirement is, thus, always fulfilled.

**Figure 4:**
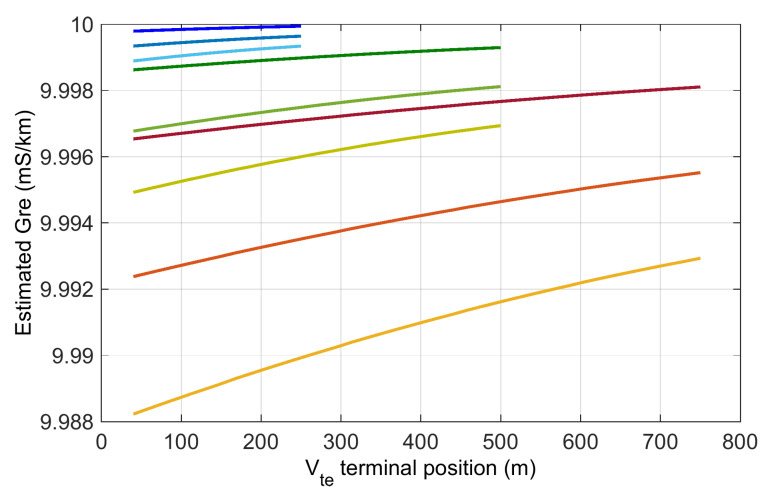
Track insulation $G_{re}$ for different rail resistance values $R_{r}=20\;m\Omega /\mathrm{km}$, 40 mΩ/km, 60 mΩ/km (shown from dark to light color) vs. voltmetric terminal position P (varying between 50 m and $L_{t}/2$ from the injection point). Track section of variable length $L_{t}=500\;m$ (blue), $L_{t}=1000\;m$ (green) and $L_{t}=1500\;m$ (red). Earthing resistance at the injection point $R_{0}=5\;\Omega$. Reference ideal value of rail insulation $G_{re}^{*}=10\;\mathrm{mS}/\mathrm{km}$.

The influence of the rail resistance was quantified in [39] by simulation, using an equivalent circuit. The variability in the track-to-earth conductance $G_{re}$ is shown in Figure 4 for a reference case $G_{re}^{*}=10\;\mathrm{mS}/\mathrm{km}$, corresponding to 100 Ωkm. The effect of rail resistance and the consequential longitudinal voltage drop is stronger for longer track sections, as expected, as the overall rail resistance is larger and, at the same time, the shunt resistance to earth is smaller. For a track length up to 1 km, the influence of the rail resistance is below 0.5% and can be made smaller by bringing the voltage terminal towards the middle of the section, rather than closer to the injection point.

After the variability sources related to the parameters and setup have been assessed, the uncertainty *per se* of (9) is evaluated straightforwardly by propagation of the uncertainty from the measured quantities for a simple V/I resistance estimate.

(10) $$u\left\{R_{te,A3}\right\}=\sqrt{{\left(u\{V_{te,A3}\left(P\right)\}\right)}^{2}+{\left(u\{I_{s}\}\right)}^{2}}$$

The expression indicates a direct dependence on the uncertainty of the voltage and current measurements that are both carried out in ideal conditions, that is, for a conveniently large value, the former, and, with a clamp (or shunt), the latter. Total instrumental uncertainty values as low as 0.5% can be easily attained; overall uncertainty may be estimated to be as low as 1% including variability, as in Figure 4, excluding the problem related to the off-potential. For the off-potential, careful selection of timing is important as the off-potential is not only taken for a large reading as track voltage, but also for the low-value readings of the rail voltage drop where even small errors are relevant (although depolarization in such readings should not take place, but fluctuation does).

## 4. Method A.4: Lateral Potential Gradient in Normal Service

This method exploits the running trains as a source of track potential fluctuations by which to estimate the track-to-earth insulation. The track is in normal condition and does not need any special arrangement; the potential is measured by connecting one conductor not interfering with the dynamic train gabarit (so, with no impact on traffic and safety).

### 4.1. Method Description and Setup

This method is well-suited for open areas where the track runs at grade without continuous civil structures to use as a potential reference, as may occur in urban and suburban at-grade line sections.

In this case a remote potential reference is taken by means of a vertical electrode driven in the soil and the measurement of the current dispersion and consequential field gradient in the soil is local, not distributed along the track section as we have considered so far for the two previous methods.

**Figure 5:**
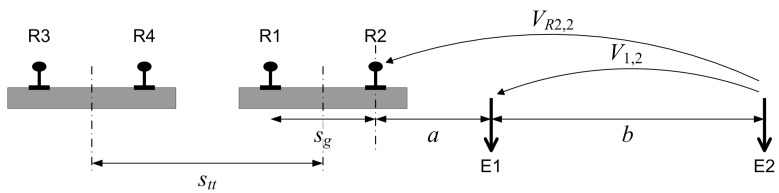
Sketch of the method A.4 setup for a track insulation measurement, showing the double track and the two electrodes (E1 and E2) and related geometrical quantities.

The method is described in Figure 5 that provides a sketch of the setup and the most relevant quantities. The setup focuses on a transversal section of the line assumed to be of negligible longitudinal size, but minor contributions from adjacent track sections cannot be ruled out. EN 50122-2 and the technical literature do not provide any indications on this to the authors’ knowledge.

The two rails of an assumed single-track layout are separated by the quantity *s* (with good approximation corresponding to the track gauge, that is, in reality, measured between the internal edges of the rail heads); the two external electrodes E1 and E2 are located in natural soil at distances *a* and a+b from the nearest rail, with *b* being the separation of the two electrodes.

The basic equation for the determination of the track-to-earth conductance $G_{TE}$ is based on the assumption of an inverse dependency with distance for the electric field in the soil.

(11) $$G_{\mathrm{TE},A4}=\frac{m_{sr}\;\pi \;1000}{\rho \left[\log(b+0.5s)-\log(a+0.5s)\right]}$$

where $m_{sr}$ is called the “stray current transfer ratio”, the factor “1000” adjusts for expressing the conductance per km, and ρ is the soil resistivity expressed in Ωm (discussed below in Section 4.2). The quantity *s* takes the value of $s_{g}$ for single-track cases and $s_{tt}$ for double-track cases.

The 2010 version of EN 50122-2 [31] reported two different formulations for the cases of single- and double-track layout, as shown in (12) and (13), respectively, using the notation $G_{\mathrm{TE},A4,1}$ and $G_{\mathrm{TE},A4,2}$. The quantities $s_{g}$ and $s_{tt}$ stand for the track gauge and the inter-track separation (measured from the track axes), respectively. The new 2021 version [29] uses only one Equation (11) and does not make such a distinction, stating simply that, for the single-track case, the quantity $s=s_{g}$, and, for the double-track case, the overall conductance must be divided by 2, and that the quantity *s* becomes the inter-track-axes distance $s_{tt}$.

(12) $$G_{\mathrm{TE},A4,1}=\frac{m_{sr}\;\pi \;2000}{\rho \left[\log\left(b(b+s_{g})\right)-\log\left(a(a+s_{g})\right)\right]}$$

(13) $$G_{\mathrm{TE},A4,2}=\frac{m_{sr}\;\pi \;1000}{\rho \left[\log\left(\left(b+0.5s_{g}\right)\left(b+0.5s_{g}+s_{tt}\right)\right)-\log\left(\left(a+0.5s_{g}\right)\left(a+0.5s_{g}+s_{tt}\right)\right)\right]}$$

The numerical difference between this different formulation of the two versions of the standard is considered below in Section 4.3.

The quantity $m_{sr}$ is stated in the standard to be determined as the linear regression of the “rail potential gradient”, as if there is a derivative operation involved. In reality, this point is not explained well, with confusion between small letters and big letters for the same quantities and the introduction of a “delta” symbol that is not then supported by any equation nor appears in the figures.

Simply, $m_{sr}$ is the angular coefficient of the linear regression of the collected rail potentials $V_{R2,2}$ vs. the inter-electrode potentials $V_{1,2}$, using electrode E2 as reference.

The estimate must be carried out with a significant number of well-distributed samples, to avoid ill-conditioning of the linear regression estimate: in other words, a short time record with all potential readings having similar values causes indeterminacy, whereas a longer record with several train passages creates an elongated cloud of points that provides a more robust estimate.

### 4.2. Practical Factors

This method is suitable for at grade scenarios, in particular, in suburban contexts, but requires free space laterally to the track of minimum 80 m (as per the recent 2022 version of EN 50122-2, but only 30 m in the older 2010 version that was more manageable).

In addition, access to natural soil near the track to place the first electrode is also necessary. This distance *a* has no minimum specification, but the standard warns that such electrodes should be far away from pits and other metallic parts near the track that could distort the field; practically speaking, as tramway and light railway tracks often run in parallel to suburban roads, such a distance is limited to a few meters maximum or, skipping the road width, is of the order of 5 m to 10 m.

The typical context, however, includes a problem of coordination with road traffic and interference with private property (e.g., accessible soil may be located in private gardens or access granted passing through private property). The method is minimally invasive, in that it requires digging a vertical electrode of small dimensions (e.g., 0.5 m) and passing of a couple of electric wires of small cross-section (e.g., 1.5 mm^2^ for mechanical robustness).

Practical constraints as well, such as the presence of a road, a park area with asphalt, or a building, may prevent access to natural soil, and, thus, require deviation from the preferred reference values for *a* and *b*, so that knowledge of the tolerances and sensitivity of results to such changes is needed. This is verified in Section 4.3.

Soil resistivity values must be determined by a separate measurement using a four-electrode method [42]. The problems related to this quantity are many:

- accessibility of the area to place the test electrodes in a line, as prescribed by the Wenner method (four electrodes in a line, with external ones for the test current $I_{t}$ and the inner ones for the voltage reading $V_{t}$, spaced by *s*); the resulting apparent soil resistivity value can be calculated from the resistance reading $R=V_{t}/I_{t}$ as ρ=2πsR; the resistivity value refers to the depth *s*, so that, to double the probed depth, the electrodes span is doubled as well;
- often, the Schlumberger method is used instead, because it requires the movement of two electrodes only, keeping the inner ones for voltage more compact; keeping their separation *s* and calling *p* the separation between each external one and the nearest voltage electrode (with p>2s), the resistivity may be estimated again from the calculated resistance value as ρ=πp(p+s)/sR and the depth is p+s/2, deeper than the previous one; in other words, for a given target depth, the Schlumberger method is more compact and faster;
- specifically focusing on the track geometry and roads nearby, keeping *s* of the order of 1 m to 2 m, the separation *p* may increase to what is allowed by the areas nearby (e.g., 5 m to 20 m); the depth values to focus on are in this range and they should be supported by a careful analysis of the resistivity values behavior to determine abnormal distributions and lack of homogeneity;
- it is, in fact, observed that interference by other metallic/conductive buried structures is almost certain in an urban/suburban context and larger volumes of soil (going deeper) help to average the contributions.

### 4.3. Variability and Uncertainty Analysis

The variability and uncertainty issues of the method are considered from three standpoints:

- first, a practical example of an extensive test campaign carried out along a tramway line is considered in order to focus on data dispersion, determination of the linear regression slope $m_{sr}$, etc.; the results are reported in the next Section 4.4 for consistency with previous sections;
- then, formulations are analyzed for sensitivity to the parameters and to robustness to extreme situations caused by practical issues, such as issues in placing electrodes;
- last, propagation of uncertainty is calculated using the given formulations, having already evaluated the behavior for uncommon values of parameters.

The determination of $m_{sr}$ is quite robust to outliers and even to a small fraction of corrupted data, provided that the recording is long enough to have a statistically significant set of good cases representing the typical dynamics of the system. As a rule of thumb, we have, in the past, used multiples of the headway time that each correspondto single tram/train passages. Deviations are possible, but, for the purpose of the determination of $m_{sr}$, they are not relevant, unless where two trams/trains pass in front of the electrodes almost at the same time. In this case, repeated occurrences are necessary so that the recording lengths of some hours are suitable. In the examples shown in the next section, the number of samples was cut to two hours. The sampling time is not of such importance, and the 2 Hz sampling time suggested by EN 50122-2 for stray current monitoring could be used.

**Figure 6:**
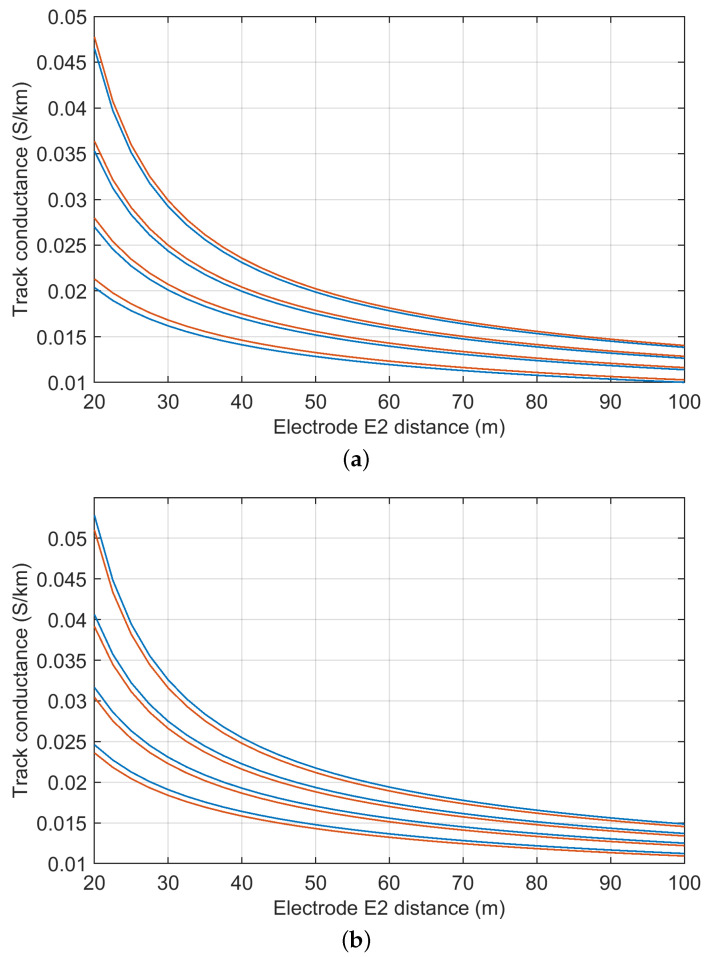
Comparison of method A4 formulas of track-to-earth conductance given in EN 50122-2 versions 2010 (blue) and 2022 (light brown): (**a**) single-track case, (**b**) double-track case. The reference parameters are: ρ=50 Ωm, $m_{sr}=0.001$, $s_{g}=1.5\;m$ and $s_{tt}=s_{g}+2\;m$. The difference between curves is of the order of 10% to 18% for the various *a* values.

EN 50122-2 has changed the two separate formulas of the 2010 version, adopting a unique formulation for both single- and double-track configurations, as introduced in Section 4.1. Figure 6 reports a comparison between formulas for single- and a double-track configurations, having fixed the inter-track separation $s_{tt}=s_{g}+2\;m$, with $s_{g}=1.5\;m$.

Considering (11), the propagation of uncertainty is operated using partial derivatives, but focusing on the quantities that are subject to the largest uncertainty ($m_{sr}$ and ρ) as the geometrical quantities *a*, *b* and *s* can be measured with high accuracy. Their uncertainty, in fact, is much less than 1% as *a* and *b* have errors lower than 1 cm over several meters and *s* is almost “exact” for mechanical and safety reasons (the rail gauge is periodically checked to be 1.435 m between the internal edges; the inter-track gap is also stable and constant as the track was positioned with accuracy of the order of mm).

(14) $$\mathrm{var}\left[G_{TE}\right]\approx \sqrt{{\left(\frac{\partial G_{TE}}{\partial m_{sr}}\right)}^{2}\mathrm{var}\left[m_{sr}\right]+{\left(\frac{\partial G_{TE}}{\partial \rho }\right)}^{2}\mathrm{var}\left[\rho \right]}$$

The two terms then correspond to:

(15) $$\frac{\partial G_{TE}}{\partial m_{sr}}=\frac{\pi \;1000}{\rho \left[\log(b+0.5s)-\log(a+0.5s)\right]}=\frac{G_{TE}}{m_{sr}}$$

(16) $$\frac{\partial G_{TE}}{\partial \rho }=-\frac{m_{sr}\;\pi \;1000}{\left[\log(b+0.5s)-\log(a+0.5s)\right]}\;\frac{1}{\rho^{2}}=\frac{G_{TE}}{\rho }$$

The uncertainty after normalization by $G_{TE}^{2}$ is then, as expected,

(17) $$u\left\{G_{TE}\right\}\approx \sqrt{{\left(u\{m_{sr}\}\right)}^{2}+{\left(u\{\rho \}\right)}^{2}}$$

Evaluating the basic uncertainty of the two quantities $m_{sr}$ and ρ is a complex task:

- For $m_{sr}$, it is a matter of propagating the uncertainty of $V_{R2,2}$ and $V_{1,2}$ through the least mean square (LMS) regression, as performed in [43] for the determination of stray capacitance (as the intercept and not the slope, as in the present case).
- For ρ, it is not a matter of uncertainty alone: the measurement itself is carried out by automatic volt-amperometric measurements at undisturbed frequencies, and the calibration with reference resistors indicates an instrumental uncertainty of the order of 1% to 2%, depending on the resistance values. The variability in the soil resistivity instead should be accounted for depending on the location, depth and environmental/seasonal conditions. The latter may be ruled out if the soil resistivity is measured immediately before (or after) the track measurements. The former can be accounted for by repeated measurements and then taking a weighted average as the ρ value and their dispersion as a Type A estimate of their uncertainty.

### 4.4. Application to a Tramway System

Method A.4 has been successfully applied to a new freshly commissioned tramway for urban sections with embedded rail that were, nevertheless, characterized by a large amount of green areas nearby (and access to natural soil). Other sections near the end of the line were instead tested during construction with method A.3, as the running rails were still not welded at several points. For the last portion of the line near the port with no access to public soil, the method A.2 was used instead over short time intervals during the day with suspension of the trial service.

**Figure 7:**
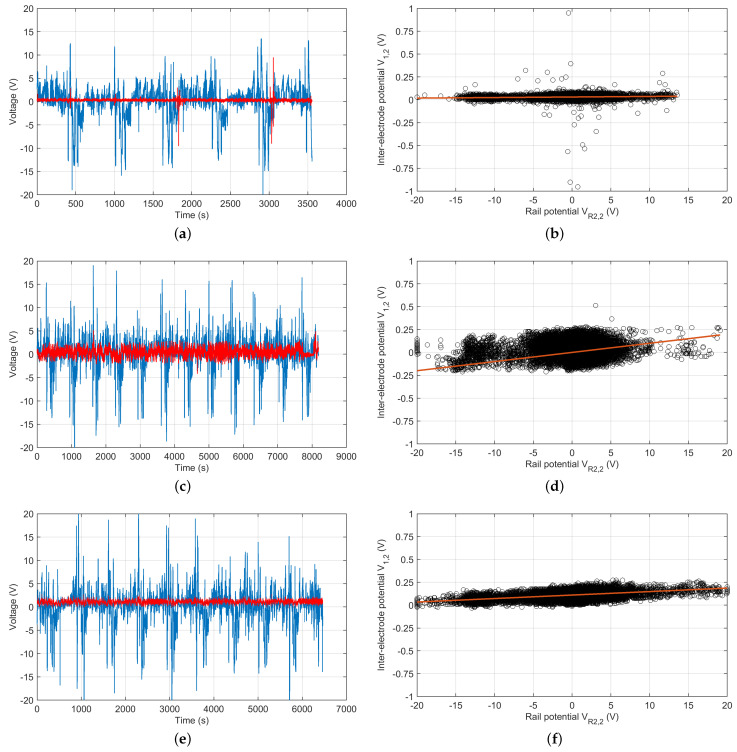
Results of method A.4 measurements for three positions along a tramway route in an urban context: (**a**,**c**,**e**) voltages of the track and electrode E1 with respect to electrode E2, in blue and red respectively; (**b**,**d**,**f**) estimated angular coefficient (stray current ratio $m_{sr}$) by linear regression (black circles are original samples, the orange line is the resulting linear regression).

Method A.4 brought with it information on track voltage values as added value. The results shown in Figure 7 report the voltages of the track and electrode E1 with respect to electrode E2 on the left and the estimated angular coefficient (stray current ratio $m_{sr}$) by linear regression on the right, providing a graphical representation of the dispersion of the data points. The orange line is the LMS regression line whose angular coefficient corresponds to $m_{sr}$: the plot of the three locations at the same vertical scale (although the $V_{R2,2}$ potential was much different), aids appreciation of the change in slope between locations due to the different values of *a* and *b*, reflecting the practical constraints of soil accessibility.

Table 2 then reports the numeric values of the estimated track conductance $G_{TE}$ and coefficient $m_{sr}$, together with the parameters of the geometry (namely, the electrode positions) and soil resistivity.

The resulting $G_{TE}$ values are quite compact, with a 4:1 proportion between the two extreme values; for an embedded track in north European climate conditions, they are quite satisfactory, being an order of magnitude below the EN 50122-2 limit of 2 S/km (embedded tramway track).

## 5. Discussion and Conclusions

This work has considered the three methods for track insulation measurement, standardized by EN 50122-2 (or IEC 62128-2). Each method has advantages and disadvantages from a system-level perspective: method A.3 requiring the electrical interruption of the running rails, in contrast to method A.2; in addition, method A.4 not only uses the track unaltered, but exploits the normal traffic as a driving signal, and is, thus, compatible with commercial service hours, in contrast to methods A.2 and A.3 that necessitate a free line and, thus, are applicable during construction or engineering hours.

In further detail, each method is based on a certain number of electrical quantities and is characterized by some level of complexity. Method A.3 is the least complex, related directly to the definition of track insulation resistance, and involves a simple volt-amperometric measurement of the track-to-earth resistance, measuring the track (or rail) voltage at an intermediate point at some distance away from the point of application of the test voltage. The uncertainty is minimal (one voltage and one current measurement), but there exists a, albeit small, variability vs. the earthing resistance of the test supply and vs. the positioning of the voltage terminal. The other two methods are, however, less invasive, not necessitating the physical sectioning of the running rails and, for method A.4, being compatible with normal traffic.

An acceptable uncertainty for track insulation assessment is never clearly made explicit in the standards and contractual specifications. Considering all the sources of variability and instrumental uncertainty, a 10% to 20% standard uncertainty level may be acceptable.

The variability and uncertainty of the methods cannot be thought of as separate, as many parameters that implicitly or explicitly are part of the track insulation equation are determined with high uncertainty (e.g., soil resistivity), are subject to change (e.g., with temperature, on a seasonal basis, etc.), or are not sufficiently constrained and depend somewhat on the operator’s choice (position of the voltage terminal, distance of the electrodes from the track, etc.). In other words, instrumental uncertainty is often a factor of lesser importance, except when the rail current is determined by voltage drop measurements (that is, the most uncertain measurement method). In this case, it was shown in Table 1 that multimeters, in general, may perform poorly if not specifically designed for such a task, e.g., mV scale reading. The accuracy of method A.2, that relies heavily on two or four rail current measurements, is, thus, significantly affected: the track length is, thus, subject to an additional constraint of minimum length to allow for a current leakage estimate with sufficient accuracy (a); such minimum length is discussed and found to be in the range of some hundreds m to 1 km, depending on the track insulation level.

Having assessed the metrological characteristics of such methods, together with other characteristics (such as the impact on system operation and the complexity of the setup), the conclusion is that methods that do not require rail sectioning should be preferred, despite their lower accuracy (including variability and uncertainty). So, research effort should be in the direction of improving repeatability and uncertainty, and, in particular, the development of methods with better spatial resolution: method A.2, in fact, is subject to the identified minimum track length requirement to preserve a minimum acceptable uncertainty level, whereas method A.4 has no clear relationship with the portion of track included in the so-determined track insulation value.

Another specific research direction is the improvement of rail current measurement, avoiding the use of the rail voltage drop, providing an immediate benefit for method A.2 in terms of uncertainty: current sensors able to measure rail current are, unfortunately, of the AC type (such as Rogowski coils and close-up magnetic sensors), so that a study should be carried out of the equivalence of DC and AC measurements, with the aim of track insulation determination for stray current assessment.

Finally, method A.4 is very promising for measurements on existing systems under normal traffic conditions (so, under real exploitation conditions) and should be further investigated in terms of the effect of the influence of buried conductive parts and the behavior of the electric field in the soil with respect to soil inhomogeneity, and, as a consequence, the required resolution and extent of soil resistivity mapping.

## Figures and Tables

**Table 1:** Examples of uncertainty values of various instruments for mV range readings.

Brand/Model	Uncert. Expression	$u\{V_{\mathrm{rX}}\}$ @ 1 mV	$u\{V_{\mathrm{rX}}\}$ @ 3 mV
Weilekes Elektronik MiniLog2	0.5% + 10 μV	1.5%	0.83%
National Instruments USB 6210	0.05% FS + 12 μV	8.9%	3.0%
Gossen Metrawatt H29S	0.02% + 0.01% FS + 5 cts.	0.02% + 0.01% 300 mV + (2 × 300 mV/30,000)/1 mV = 0.02% + 3% + 2% = 5.02%	0.02% + 0.01% 300 mV + (2 × 300 mV/30,000)/3 mV = 0.02% + 3% + 0.66% = 3.68%
Fluke 117	0.5% + 2 cts.	0.5% + (2 × 600 mV/6000)/1 mV = 20.5%	0.5% + (2 × 600 mV/6000)/3 mV = 7.17%

**Table 2:** Worked out method A4 on three locations of the same tramway system: geometry parameters and main results.

Location	ρ (Ωm)	*a* (m)	*b* (m)	$s_{g}$ (m)	$s_{\mathrm{tt}}$ (m)	$m_{\mathrm{sr}}$	$G_{\mathrm{TE}}$ (Skm)
1	17.1	11.1	37.8	1.5	6.7	0.000605	0.0436
2	19.8	14.2	46.2	1.5	3.7	0.0026	0.1525
3	38.6	8.6	45.7	1.5	3.9	0.0038	0.0949

## Data Availability

The data presented in this study are available on request from the corresponding author. The data are not publicly available due to confidentiality of method A.4 tests carried out in a specific project.
